# The complete chloroplast genome of a gynodioecious deciduous orchid *Satyrium ciliatum* (Orchidaceae) female

**DOI:** 10.1080/23802359.2019.1687359

**Published:** 2019-11-08

**Authors:** Xiaokai Ma, Han Lin, Yanqiong Chen, Siren Lan, Ray Ming

**Affiliations:** FAFU and UIUC-SIB Joint Center for Genomics and Biotechnology, The Key Laboratory of National Forestry and Grassland Administration for Orchid Conservation and Utilization (Fuzhou), College of Landscape Architecture, Fujian Agriculture and Forestry University, Fuzhou, China

**Keywords:** *Satyrium ciliatum*, gynodioecy, plastid genome, phylogeny, orchid

## Abstract

The chloroplast (cp) genome sequence of a gynodioecious *Satyrium ciliatum* female type has been characterized using Illumina pair-end sequencing. The complete cp genome was 154,418 bp in length, containing a large single copy region (LSC) of 83,475 bp and a small single copy region (SSC) of 17,513 bp, which were separated by a pair of 26,715 bp inverted repeat regions (IRs). The genome contained 132 genes, with 113 unique genes, including 79 protein-coding genes, 30 tRNA genes, and 4 rRNA genes. The overall GC content is 37.18% with the values of the LSC, SSC, and IR regions are 34.90%, 30.15%, and 43.06%, respectively. Phylogenetic analysis suggested that the *S. ciliatum* is close to *Platanthera japonica* (MG925368) in subfamily Orchidoideae.

*Satyrium ciliatum* Lindl., is a deciduous subalpine species native to southern China (Chen et al. 2009). The gynodioecious sexual system contains two floral phenotypes, with one female (male sterility) and one hermaphrodite, which is the model to study the sex determination in orchids (Huang et al. 2009). Moreover, the phylogenetic position of *S. ciliatum* in Orchidoideae subfamily is still unresolved. In this study, we first reported the complete chloroplast genome of *S. ciliatum* female and its phylogenetic position in the subfamily Orchidoideae of orchids.

The leaf samples of *S. ciliatum* female were collected from Kunming, Yunnan, China (N 26°17′, E 103°01′) and dried in liquid nitrogen. The specimen was deposited in Fujian Agriculture and Forestry University (specimen voucher X.K. Ma 006). The total genomic DNA was extracted using a DNA extraction kit (Tiangen Biotech Ltd. Beijing, China). DNA was sequenced (pair-end 150 bp reads) on an Illumina Hiseq 2500 platform. Totally 10 Gb raw reads were generated. The filtered reads were assembled using GetOrganelle pipe-line (https://github.com/Kinggerm/GetOrganelle) referring to a list of complete chloroplast genomes in Orchidaceae. The assembled cp genome was viewed and edited using Bandage (Wick et al. 2015), and then was annotated using Geneious R11.15 (Biomatters Ltd., Auckland, New Zealand) (Kearse et al. 2012). OGDRAW (http://ogdraw.mpimp-golm.mpg.de/) (Lohse et al. 2013) was used to visualize the physical map and annotations.

The annotated complete chloroplast genome was submitted to GenBank with accession NO. MN497244. The plastome of *S. ciliatum* female is 154,418 base pairs (bp) in length, containing a large single-copy (LSC) region of 83,475 bp, and a small single-copy (SSC) region of 17,513 bp, which were separated by two inverted repeat (IR) regions of 26,715 bp. It possesses total 132 genes, with 113 unique genes (including 79 protein-coding genes, 30 tRNA genes, and 4 rRNA genes). Among all of these genes, six protein-coding genes (i.e. *ndh*B, *rp*l2, *rpl*23, *rps*7, *rps*19, *ycf*2), four rRNA genes (i.e. 4.5S, 5S, 16S, and 23S rRNA), and eight tRNA genes (i.e. *trn*A-UGC, *trn*H-GUG, *trn*I-CAU, *trn*I-GAU, *trn*L-CAA, *trn*N-GUU, *trn*R-ACG, *trn*V-GAC) occur in double copies, one protein-coding gene (*rps*12) occurs in three copies. The overall GC-content of the whole plastome is 37.18%, with values of 34.90%, 30.15%, and 43.06% respectively in LSC, SSC, and IR regions.

To understand the phylogenetic position of *S. ciliatum* female, a molecular phylogenetic tree was constructed based on the cp genomes of five other species in subfamily Orchidoideae and two outgroup species in subfamily Cypripedioideae using IQ-tree (Nguyen et al. 2015) with 1000 bootstrap replicates. All eight sequences were aligned using Muscle (Edgar 2004) and then were pruned using BMGE tools (Criscuolo and Gribaldo 2010). The best nucleotide substitution model TVM + F+R2 was chosen to construct the phylogenetic tree according to BIC criteria using IQ-tree. Our phylogenetic result showed that the *S. ciliatum* female is close to *Platanthera japonica* (MG925368) with 100% bootstrap support (Figure 1). This newly reported chloroplast genome provides a good foundation for the identification of *Satyrium* species, constructing the phylogenetic relationships among Orchidoideae subfamily and studying their plastome evolution.

**Figure 1.**
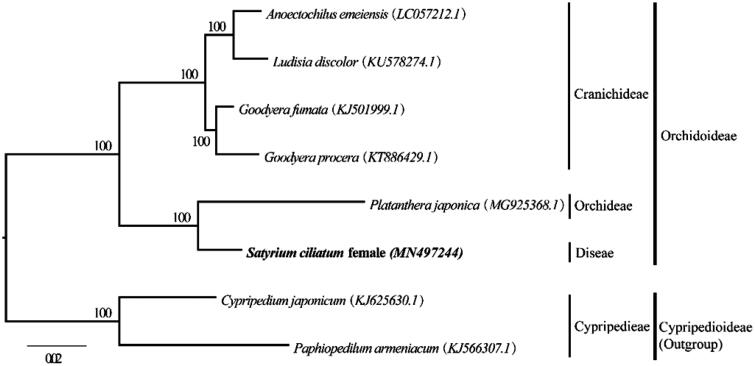
Phylogenetic position of *Satyrium ciliatum* female inferred by maximum-likelihood (ML) tree based on six complete cp genome in subfamily Orchidoideae, with two species *Cypripedium japonicum* (KJ625630) and *Paphiopedilum armeniacum* (KJ566307) in subfamily Cypripedioideae as outgroups. The bootstrap support value with 1000 replicates labeled on each node.

## References

[CIT0001] Chen X, Liu Z, Zhu GH, Lang KY, Ji ZH, Luo YB, Jin XH, Cribb PJ 2009 Orchidaceae, flora of China. Science Press, Beijing & Missouri Botanical Garden Presss, St. Louis; p. 260

[CIT0002] Criscuolo A, Gribaldo S 2010 BMGE (Block Mapping and Gathering with Entropy): a new software for selection of phylogenetic informative regions from multiple sequence alignments. BMC Evol Biol. 10(1):210. 20626897 10.1186/1471-2148-10-210 PMC3017758

[CIT0003] Edgar RC 2004 MUSCLE: multiple sequence alignment with high accuracy and high throughput. Nucleic Acids Res. 32(5):1792–1797. 15034147 10.1093/nar/gkh340 PMC390337

[CIT0004] Huang SQ, Lu Y, Chen YZ, Luo YB, Delph LF 2009 Parthenogenesis maintains male sterility in a gynodioecious orchid. Am Nat. 174(4):578–584. 19689212 10.1086/605378

[CIT0005] Lohse M, Drechsel O, Kahlau S, Bock R 2013 Organellar genome DRAW-a suite of tools for generating physical maps of plastid and mitochondrial genomes and visualizing expression data sets. Nucleic Acids Res. 41:W575–W581. 23609545 10.1093/nar/gkt289 PMC3692101

[CIT0006] Kearse M, Moir R, Wilson A, Stones-Havas S, Cheung M, Sturrock S, Buxton S, Cooper A, Lohse M, Drechsel O, et al. 2012 Geneious basic: an integrated and extendable desktop software platform for the organization and analysis of sequence data. Bioinformatics. 28:1647–1649. 22543367 10.1093/bioinformatics/bts199 PMC3371832

[CIT0007] Nguyen LT, Schmidt HA, von Haeseler A, Minh BQ 2015 IQ-TREE: a fast and effective stochastic algorithm for estimating maximum-likelihood phylogenies. Mol Biol Evol. 32(1):268–274. 25371430 10.1093/molbev/msu300 PMC4271533

[CIT0008] Wick RR, Schultz MB, Zobel J, Holt KE 2015 Bandage: interactive visualization of de novo genome assemblies. Bioinformatics. 31(20):3350–3352. 26099265 10.1093/bioinformatics/btv383 PMC4595904

